# Dielectric relaxation, resonance and scaling behaviors in Sr_3_Co_2_Fe_24_O_41_ hexaferrite

**DOI:** 10.1038/srep13645

**Published:** 2015-08-28

**Authors:** Rujun Tang, Chen Jiang, Wenhu Qian, Jie Jian, Xin Zhang, Haiyan Wang, Hao Yang

**Affiliations:** 1 Jiangsu Key Laboratory of Thin Films, College of Physics, Optoelectronics and Energy, Soochow University, Suzhou 215006, P. R. China; 2 Testing and Analysis Center, Soochow University, Suzhou 215006, P. R. China; 3 Department of Electrical and Computer Engineering, Texas A&M University, College Station, Texas 77843-3128, USA; 4 School of Materials Science and Engineering, Guilin University of Electronic Technology, Guilin 541004, P. R. China; 5 College of Science, Nanjing University of Aeronautics and Astronautics, Nanjing 211106, P. R. China

## Abstract

The dielectric properties of *Z*-type hexaferrite Sr_3_Co_2_Fe_24_O_41_ (SCFO) have been investigated as a function of temperature from 153 to 503 K between 1 and 2 GHz. The dielectric responses of SCFO are found to be frequency dependent and thermally activated. The relaxation-type dielectric behavior is observed to be dominating in the low frequency region and resonance-type dielectric behavior is found to be dominating above 10^8^ Hz. This frequency dependence of dielectric behavior is explained by the damped harmonic oscillator model with temperature dependent coefficients. The imaginary part of impedance (*Z*″) and modulus (*M*″) spectra show that there is a distribution of relaxation times. The scaling behaviors of *Z*″ and *M*″ spectra further suggest that the distribution of relaxation times is temperature independent at low frequencies. The dielectric loss spectra at different temperatures have not shown a scaling behavior above 10^8^ Hz. A comparison between the *Z*″ and the *M*″ spectra indicates that the short-range charges motion dominates at low temperatures and the long-range charges motion dominates at high temperatures. The above results indicate that the dielectric dispersion mechanism in SCFO is temperature independent at low frequencies and temperature dependent at high frequencies due to the domination of resonance behavior.

Hexaferrites are iron oxides with hexagonal structures and have long been used in technological applications such as permanent magnets and microwave devices^1^. The Z-type hexaferrites (Ba,Sr)_3_Me_2_Fe_24_O_41_ (Me is a divalent transition metal ion) are one of the families of hexagonal ferrites. The crystal structure of Z-type hexaferrite can be described as alternating stacking of the basic blocks RSTSR*S*T*S* (the asterisk corresponds to an 180° block turning around the hexagonal *c-*axis) where S is (Ba,Sr)Fe_2_O_4_, R is [(Ba,Sr)Fe_6_O_11_]^2−^ and T is (Ba,Sr)_2_Fe_8_O_14_. Recently, it was reported that the polycrystalline Z-type ferrite Sr_3_Co_2_Fe_24_O_41_ could exhibit low-field magnetoelectric effects at room temperature^2^,^3^,^4^. The magnetic field tuning of electric polarization in Sr_3_Co_2_Fe_24_O_41_ was found to be induced by its transverse conical spin structure through the inverse Dzyaloshinskii-Moriya mechanism^3^. It was further revealed that the single crystalline Sr_2.48_Ba_0.52_Co_2_Fe_24_O_41_ Z-type ferrite exhibits a magnetoelectric susceptibility of 3200 ps/m which was much higher than other room temperature single phase multiferroic materials, for example BiFeO_3_ (55 ps/m)^5^,^6^. The findings of the room temperature magnetoelectric effects in Z-type hexaferrites will enable this material to be applied in the novel low-power magnetoelectronic devices^7^,^8^.

For the magnetoelectronic devices operating in the microwave region or under an alternative electric field, both electrical and magnetic properties of the device materials are important for the devices’ performance. Although the magnetic and magnetoelectric properties of the Z-type hexaferrites have been intensively studied in the recent years^2^,^3^,^4^,^5^,^7^,^8^,^9^,^10^,^11^, only few works have been reported on the dielectric properties of Z-type hexaferrites. The composition dependent dielectric properties of Z-type hexaferrite Ba_3_Co_2_Fe_24_O_41_ have been reported^12^,^13^,^14^. But the dielectric properties of substituted Ba_3_Co_2_Fe_24_O_41_ were investigated only at room temperature above 10^7^ Hz. In addition, the impedance properties of Ba_3_Co_2_Fe_24_O_41_ have not been discussed. A recent work reported the observation of a large negative magnetodielectric effect of Sr_3_Co_2_Fe_24_O_41_ (SCFO) hexaferrite below 10^5^ Hz^9^. However, the dielectric properties of SCFO have not been detailed discussed in this work. In addition, a systematical investigation on the temperature dependence of dielectric properties of SCFO has not been reported up to now^2^,^3^,^4^,^5^,^7^,^8^,^9^. The above works show that the reported studies on the dielectric properties of Z-type hexaferrites are still very limited. The dielectric dispersion mechanism and the microstructure-electrical property relationship in the Z-type hexaferrites, which is both physically interesting and technically important, have not been well understood until now. In this work, the dielectric permittivity, impedance and modulus of SCFO have been investigated as a function of the frequency (1–2 G Hz) and temperature (153 K–503 K). Both the relaxation-type and resonance-type dielectric behaviors in SCFO are observed. A damped harmonic oscillator model is used to explain the frequency dependence of dielectric behaviors at different temperatures. In addition, the scaling behaviors of imaginary impedance and modulus spectra are observed at low frequencies.

## Results

### Characterization of SCFO polycrystalline sample

**Figure 1:**
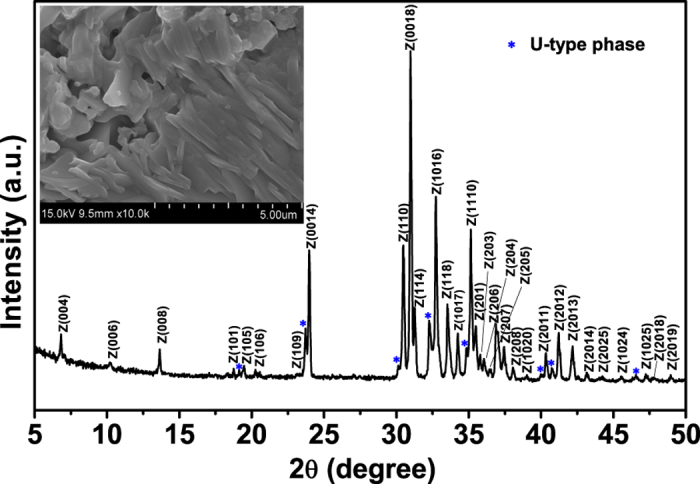
XRD pattern of the SCFO sample taken at room temperature, the Z type phase has been indexed according to the calculated spectrum. The inset is the SEM image of the sample.

Figure 1 is the X-ray diffraction *θ*–2*θ* spectrum of the sample. The peaks have been indexed according to the calculated Z-type SCFO spectra in the references^2^,^15^. Figure 1 shows that the majority phase of the sample is Z-type SCFO. However, a small amount of impurity U-type hexaferrite (Sr_4_Co_2_Fe_36_O_60_) exists in the sample. It has been shown that preparation of the pure Z-type phase is extremely difficult because other hexaferrite phases usually coexist with the Z-type during the synthesis process^2^,^3^,^5^,^7^,^9^. It can be expected that the electrical responses of the Z-type and U-type (sequence RSR*S*T*S*) phases are similar because they have the same stacking blocks and very close stacking sequences. Therefore, the dielectric properties of the polycrystalline sample in this work can’t be substantially changed by the existence of the U-type impurity^2^. The grain structure of SCFO was measured with Scanning Electron Microscope as shown in the inset of Fig. 1. It can be seen that the SCFO sample has plate-like grains which stack on each other.

**Figure 2:**
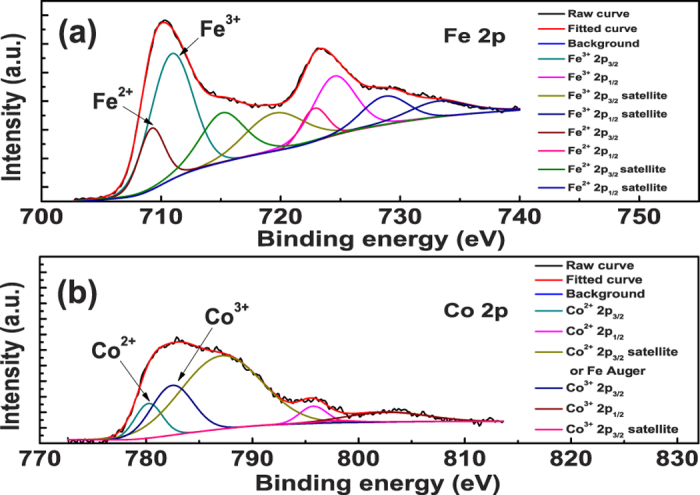
XPS patterns of (a) the Fe ions and (b) Co ions in SCFO at room temperature.

Figure 2 shows the X-ray photoelectron spectroscopy (XPS) spectra of the magnetic ions of the SCFO sample. Figure 2 shows that both divalent ions (Fe^2+^ and Co^2+^) and trivalent ions (Fe^3+^ and Co^3+^) exist in the sample. The electronic exchange between Fe^2+^ and Fe^3+^ produces *n*-type carriers and also the hole exchange between Co^3+^ and Co^2+^ gives rise to *p*-type carriers^12^,^13^,^16^. The formation of both types of charge carriers arises from the loss of oxygen during the high temperature sintering process. For charge compensation, a part of Fe^3+^ transforms to Fe^2+^ and a part of Co^2+^ transforms to Co^3+^. All the carriers involved in the sample will affect the dielectric dispersion behavior. However, it can be expected that the electron exchange between Fe^3+^ and Fe^2+^ will be dominating in SCFO due to the large atomic ratio of Fe in SCFO^12^,^13^.

### Dielectric permittivity analysis

**Figure 3:**
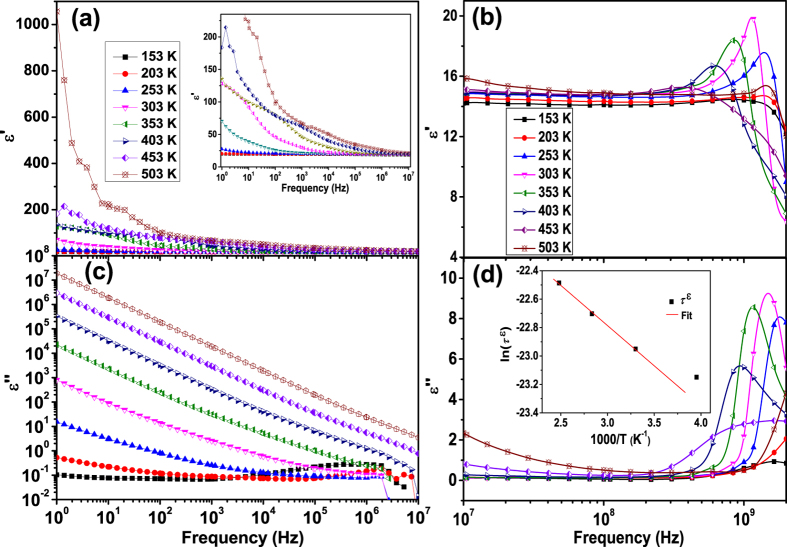
Temperature dependent dielectric constant ε′ spectra (a) below 10^7^ Hz and (b) above 10^7^ Hz, dielectric loss ε″ spectra (c) below 10^7^ Hz and (d) above 10^7^ Hz, the inset of plot (d) is the temperature dependent relaxation times obtained from the peak frequencies of ε″spectra between 253 K and 403 K.

Figure 3 shows the frequency dependent dielectric constant *ε*′ and dielectric loss *ε*″ at different temperatures. Figure 3 shows that when the temperature is lower than 203 K, both *ε*′ and *ε*″ are rather small and have very weak dependences on the frequency below 1 GHz. This phenomenon has been referred as the “nearly constant loss” (NCL) behavior^17^,^18^. The NCL is generally regarded as resulting from relaxations of the charges moving in the asymmetric double-well potentials^19^. Such relaxation involves highly localized motions rather than the dominating hopping processes which usually occur in the ferrites at high temperatures^12^,^13^,^16^,^20^,^21^,^22^. When the temperature is increased above 253 K and the frequency is lower than 10^8^ Hz, both *ε*′ and *ε*″ decrease with increasing frequency and increase with increasing temperature generally. The above frequency and temperature dependent *ε*′ and *ε*″ can be explained by Koop’s theory which takes the dielectric structure as an inhomogeneous medium composed of two Maxwell-Wagner type layers^23^,^24^,^25^. In this model, the dielectric structure is imagined to consist of fairly well-conducting ferrite grains separated by poorly conducting grain boundaries. The electrons reach the grain boundaries through hopping and if the resistance of grain boundaries is high enough, electrons pile up at the grain boundaries and produce polarization. However, as the frequency of the applied field is increased, the electrons reverse their direction of motion more often. This decreases the probability of electrons reaching the grain boundary and as a result, polarization decreases. Therefore, both *ε*′ and *ε*″ decrease with an increase in frequency. The decrease of *ε*″ is due to the decreased conductive process in the sample. The previous XPS results in Fig. 2 show that there are both iron and cobalt ion defects in SCFO which produce *n*-type and *p*-type carriers respectively. The electrons contribute to polarization by local displacement in the direction opposite to the electric field while the holes contribute to polarization by local displacement in the direction of the external electric field. The collective contribution of both types gives the dielectric response. With an increase in temperature, the electron hopping between Fe^2+^ and Fe^3+^ and hole hopping between Co^3+^ and Co^2+^ are thermal activated. This increases the probability of electrons reaching the grain boundary and as a result, polarization increases. Therefore, both *ε*′ and *ε*″ increase with increasing temperature.

When the frequency is higher than 10^8^ Hz, strong peaks are observed in both *ε*′ and *ε*″ spectra above 0.5 GHz between 203 K and 453 K. Both the *ε*′ peaks and *ε*″ peaks shift toward lower frequencies with increasing temperature. With an increase in temperature, the peak intensity increases firstly with a maximum value at 303 K and then decreases. In addition, the peak broadens with an increased asymmetry as the temperature increases. This shows again that the dielectric response at high frequency is temperature dependent. Moreover, the simultaneous appearance of *ε*′ and *ε*″ peaks with a slightly higher *ε*″ peak position has been referred as “anomalous dispersion” phenomenon^26^. In this case, the dielectric response is dominated by the resonance behavior instead of the relaxation behavior which usually occurs in the low frequency regions^26^,^27^,^28^.

The frequency dependence of complex dielectric constant can be described by a damped harmonic oscillator model with the expression^26^,^27^,^28^,^29^


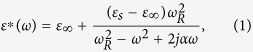


Then, the real and imaginary parts of the complex dielectric constant can be written as


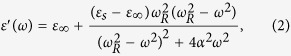



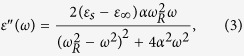


where *ε*_*s*_ and *ε*_∞_ are the static- and high-frequency limits of the dielectric constant, respectively, *ω = *2*πf* is the angular frequency, *ω*_*R*_ is the temperature dependent natural frequency of oscillation of the harmonic oscillator in the absence of damping, and *α* is the temperature dependent damping coefficient of the dielectric response. The parameter *k* =2*α*/*ω*_*R*_ is an essential parameter for identifying the dielectric response mechanism. When *k* is zero, only resonance-type dielectric response takes place in the dielectric system in which the charge moving is dominated only by the inertial effects^26^. In this case, the dielectric loss is a delta function with an infinitely large value. When *k* is larger than zero, relaxation-type dielectric response comes into play. With an increase in *k*, the dielectric response of the system will pass from a resonance-dominated behavior (*k* < 1) to a relaxation-dominated behavior gradually and finally, to the Debye-type behavior which corresponds to a pure relaxation-type dielectric behavior with infinitely strong damping. In addition, the dielectric loss peak will broaden with increasing *k*^26^.

**Figure 4:**
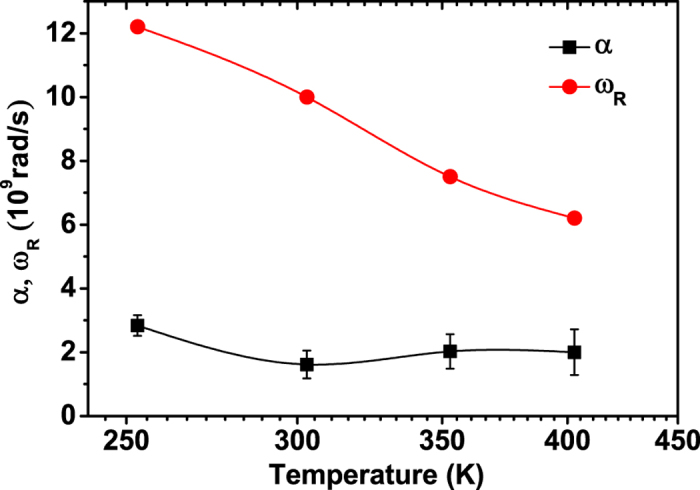
Temperature dependence of the damping coefficient *α* and peak frequency *ω*_*R*_ of the dielectric loss, the *α* is obtained from the least-square fitting of dielectric loss ε″ spectra above 0.2 GHz to the Eq. (3), the error bar is the fitting standard error.

The high frequency dielectric loss peaks in Fig. 3(d) are fitted with Eq. (3) with *α* and Δ*ε* = *ε*_*s*_ − *ε*_∞_ as the fitting parameters. *ω*_*R*_ has been calculated with the relation 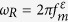 where 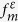 is peak frequency of *ε*″. The fitting results of *α* and *ω*_*R*_ at different temperatures are shown in Fig. 4. Figure 4 shows that *α* is much smaller than *ω*_*R*_ (*k *= 2*α*/*ω*_*R*_ < 0.65) at all temperatures, indicating that the electrical damping in SCFO is small. This agrees with the appearance of resonance peaks in the permittivity spectra. In addition, with an increase in temperature, the fitting standard error increases due to the increased asymmetry of the peaks. It must be mentioned that Eq. (1) is derived from the damped motions of many identical non-interacting dipole oscillators. In the real sample, the dipole interactions are inevitable. In addition, it has been shown in Fig. 2 that there are both *n*-type and *p*-type carriers and hence different dipole oscillators which may have different temperature dependences in the SCFO sample. The above two factors and the electrical non-uniformities in the polycrystalline sample may lead to an increased peak width and asymmetry of the loss peak. An increase in temperature may lead to a variation of both charge carrier densities and dipole interactions^28^. Therefore, the deviation of the experimental data from the ideal damped harmonic oscillator model increases with increasing temperature. However, we can still see clearly from Fig. 4 that the ratio between *α* and *ω*_*R*_ (*k* = 2*α*/*ω*_*R*_) increases with increasing temperature despite of the increase in fitting deviations. This indicates that increasing temperature leads to an increased damping motion of the dipoles and thus increased dielectric relaxation behaviors of the sample. This can be further evidenced by the decreased peak intensities on both *ε*′ and *ε*″ spectra with increasing temperature as shown in Fig. 3(b,d). The above results show that the high frequency dielectric response of the sample is firstly thermal activated with increasing temperature and then damped with a further increase in temperature.

The most probable dielectric relaxation time *τ*^*ε*^ of the sample is calculated using the relation 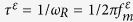. The *τ*^*ε*^ as a function of temperature is shown in the inset of Fig. 3(d). Result shows that *τ*^*ε*^ increases with increasing the temperature, which agrees with the increase in the damping strength in the previous discussions. In addition, the *τ*^*ε*^ above 303 K can be well fitted with the Arrhenius relation 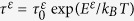, where 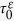 is the pre-exponential factor of the relaxation time, *T* is the temperature, and *k*_B_ is the Boltzmann constant. The activation energy 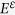 determined from the slope of the linear fit in the Arrhenius plot is 0.05 eV which is much lower than the values obtained from the dielectric relaxation peaks at low frequencies for many ferrites^22^,^30^,^31^,^32^. This agrees with the small damping dielectric behavior above 0.5 GHz in the SCFO sample.

**Figure 5:**
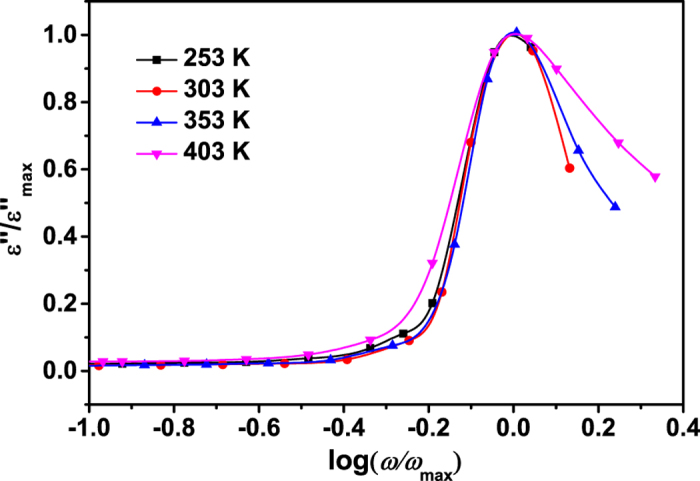
The dielectric loss ε″ spectra plotted with scaled coordinates above 10^8^ Hz at different temperatures.

The above permittivity spectra shows that the dielectric response is non-Debye type and temperature dependent in the high frequency region. In order to confirm whether the distribution of relaxation times is temperature dependent or not, we plotted the *ε*″ in scaled coordinates for the spectra above 10^8^ Hz, i.e., 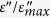 versus log(*ω*/*ω*_*max*_), where 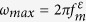 is the loss peak frequency. If all the permittivity loss profiles are collapsed into one master curve, it suggests that the distribution of relaxation times is temperature independent^33^. Figure 5 shows that for the permittivity spectra at temperature below 353 K, the peaks are nearly overlapped in relatively low frequency region. With a further increase in frequency or an increase in temperature to 403 K, the dispersion of the spectra occurs. The above results indicate that the distribution of the relaxation times is temperature dependent in the frequency region above 10^8^ Hz.

### Complex impedance analysis

**Figure 6:**
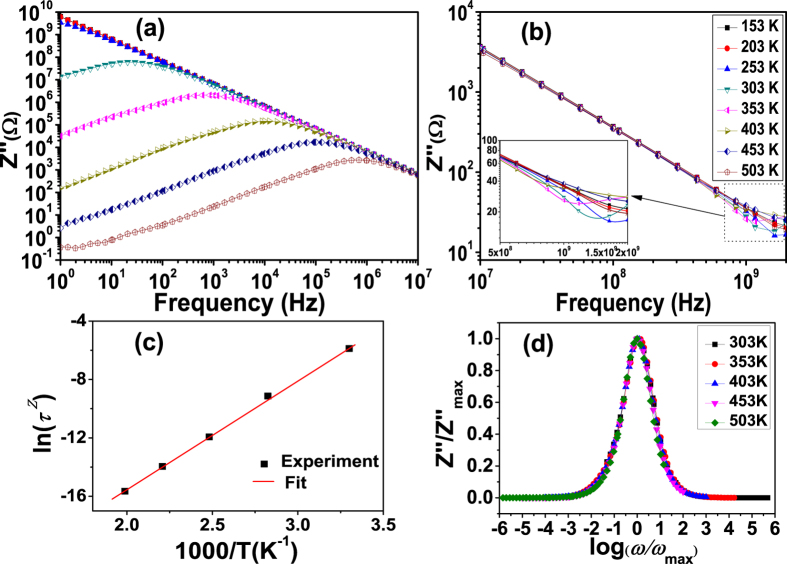
Temperature dependent imaginary part impedance *Z*″ spectra (a) below 10^7^ Hz and (b) above 10^7^ Hz, (c) the temperature dependence of the relaxation times obtained from the peak frequencies of *Z*″spectra above 303 K, (d) scaling behavior of the *Z*″ spectra in plot (a) at temperatures above 303 K.

In order to get better understanding on the dielectric relaxation behaviors of SCFO, the impedance and modulus spectra of the sample have been studied. Figure 6 shows the frequency dependence of imaginary part (*Z*″) of the impedance Z^*^ = *Z*′ − *iZ*″at different temperatures. Figure 6(a,b) show that at temperatures below 303 K, *Z*″decreases monotonically with increasing frequency and merges together at a very low value when frequency exceeds 100 Hz. On the other hand, the magnitudes of *Z*″ decrease with increasing temperature. The monotonic decrease of *Z*″ with increasing frequency indicates that at low temperatures, the relaxation is very weak. This should be due to the freezing of dipoles at low temperatures as indicated by the small values of dielectric constant and dielectric loss at low temperatures in Fig. 3. When the temperature is increased above 303 K, a strong impedance loss peak appears in the measured frequency range. The loss peak moves to higher frequency with a fall in intensity as the temperature increases. This implies that electrical responses are thermally activated. This is possibly due to the presence of space charge at the grain boundaries^12^,^13^,^14^. The shifting of peak position to higher frequency with increasing temperature indicates an increase in the relaxation rate. The dielectric relaxation time *τ*^*Z*^ is calculated using the relation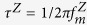, where 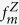 is peak frequency of *Z*″. This relaxation time as a function of temperature is shown in Fig. 6(c). As the temperature increases, the relaxation time decreases, which shows an opposite tendency of temperature dependence to that in Fig. 3(d). This should be attributed to the semiconductor-like conduction nature of the samples. It can be seen that the relaxation time *τ*^*Z*^ can be well fitted with the Arrhenius relation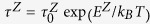, where 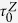 is the pre-exponential factor of the relaxation time. The activation energy *E*^*Z*^ determined from the slope of the linear fit in the Arrhenius plot is 0.66 eV which is much higher than the value (0.05 eV) obtained from the *ε*″ spectra. This indicates again that the dielectric relaxation behavior undergoes a higher damping strength than the dielectric resonance behavior.

Moreover, the full width at half maxima calculated from the impedance loss spectra (*Z*″ vs log *f*) are greater than 1.144 decades (ideal Debye relaxation), which indicates the deviation from Debye-type relaxation^34^. This deviation from ideal Debye relaxation might be due to an inherently nonexponential process, such as correlation between diffusive motion of the charge carriers, or nonuniformities in the material microstructure, leading in turn to a spatial distribution of local conductivities and electrical response times^34^,^35^,^36^. In addition, the *Z*″ curves at all temperatures merge together at high frequencies. The merge of the *Z*″ at higher frequency indicates a possible release of space charges at the grain boundaries and therefore, the high frequency dielectric response are dominated by the electrical response within the crystalline grains. Figure 6(b) show that when the frequency is increased above 0.5 GHz, dispersions of *Z*″spectra appear again. This should be due to the resonance-dominated dielectric behavior in the high frequency region as shown in Fig. 3.

The above impedance spectra shows a poly-dispersive nature for the dielectric relaxation at low frequencies. In order to confirm whether the distribution of relaxation times is temperature dependent or not, we plotted the *Z*″ in scaled coordinates, i.e., 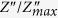 versus log(*ω*/*ω*_*max*_), where 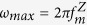 is the loss peak frequency. If all the impedance loss profiles are collapsed into one master curve, it suggests that the distribution of relaxation times is temperature independent^34^. Figure 6(d) shows that all peaks indeed overlap into one master curve at different temperatures above 303 K. It suggests that the dynamic processes of the charges occurring at different time scales exhibit the same activation energy and that the distribution of the relaxation times is temperature independent.

**Figure 7:**
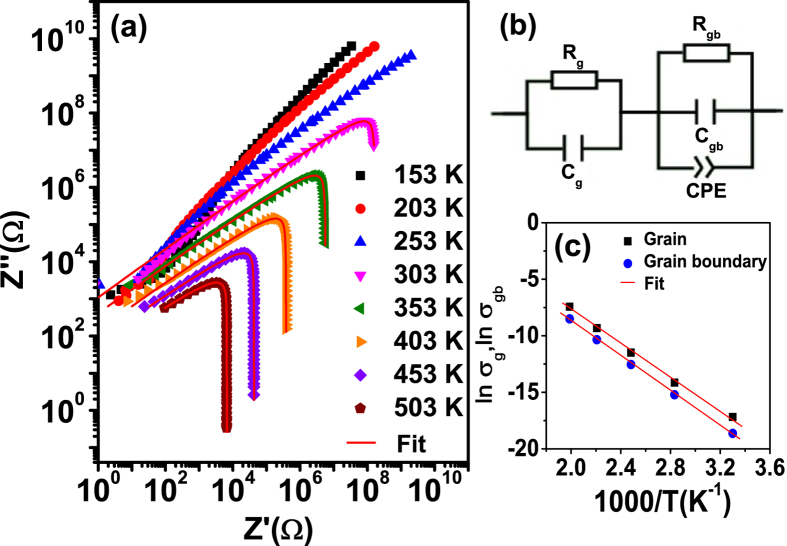
(**a**) Log-log plots of complex impedance (*Z*′ vs *Z*″) at different temperatures, (**b**) equivalent circuit based on the brick-layer model for the impedance spectra, (**c**) Arrhenius plots of the *dc* conductivities for grain and grain boundary obtained from the fitting results of equivalent circuit above 303 K.

Figure 7 shows the complex impedance plane plots (*Z*′ vs *Z*″) and their fitting results. In order to compare the two responses with vastly different impedances in one plot, the log-log presentation of the complex impedance plots are presented. Although the semicircular arcs are distorted, logarithmic plot still offers significant advantages in several respects which have been discussed by Jonscher^26^. Figure 7(a) shows that there is only one arc appears in the right side of the curve which corresponds to the semicircular arc in the linear complex impedance plot. Furthermore, the arcs shift from right to left with reduced diameters as the temperature increases due to the associated decreases of the impedances.

In order to analyze the impedance data and establish a connection between microstructure and electrical properties, data are usually modeled by an ideal equivalent electrical circuit comprising of resistance (*R*) and capacitance (*C*). The polycrystalline materials generally show both grain and grain-boundary impedances. We find that the impedance data above 303 K in Fig. 7(a) can be best fitted with the equivalent circuit based on the brick-layer model as shown in Fig. 7(b)^37^. This circuit consists of a series array of two sub-circuits, one represents grain effects and the other represents grain boundaries. Let (*R*_g_, *R*_gb_) and (*C*_g_, *C*_gb_) represent the resistances and capacitances of grains and grain boundaries, respectively, and *CPE* denotes a constant phase element indicating the departure from ideal Debye-type model. The *CPE* admittance is *Y*_*CPE*_ = *A*_0_(*jω*)^*n*^, where *A*_0_ and *n* are parameters depending on temperature only, *A*_0_ confines the magnitude of the dispersion and 0 < *n* < 1. The parameter of *n* is equal to 1 for ideal capacitor and equal to 0 for ideal resistor^37^. Hence, the equation for the equivalent circuit in Fig. 7(b) can be represented by:


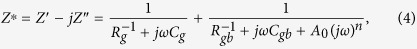


where,


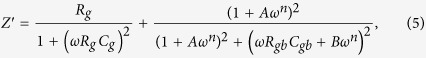



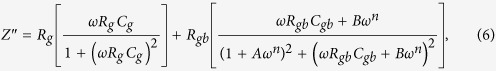


in which


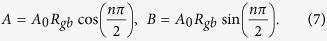


Our data were then fitted using software *ZSIMPWIN* version 2 by assuming the equivalent circuit discussed above. The impedance data below 303 K are not taken into the fitting because it is not a precise way to fit the low temperature data by extrapolating these straight lines to semicircular arcs. The fitted values of *n* of *CPE* at temperatures above 303 K are in the range of 0.47–0.63 and increase with increasing temperature. Figure 7(c) shows the fitted dc conductivities for grain *σ*_*g*_(*σ*_*g*_ ∝1/*R*_*g*_) and grain boundary *σ*_*gb*_ (*σ*_*gb*_∝1/*R*_*gb*_) plotted against the reciprocal temperature in the Arrhenius format. The fitting results indeed show that *σ*_*gb*_ is smaller than *σ*_*g*_. This should be due to the lower concentration of oxygen vacancies and trapped electrons in grain boundaries during the cooling re-oxidation process^37^,^38^. The difference between *σ*_*g*_and *σ*_*gb*_ will lead to the piling up of space charges at the grain boundaries and producing polarization (Maxwell-Wagner effect)^16^,^22^,^31^,^32^.

Figure 7(c) also shows that both *σ*_*g*_ and *σ*_*gb*_ obey the Arrhenius law *σ* = *σ*_0_ exp(*E*^*Z*^/*k*_*B*_*T*), where *σ*_0_ is the prefactor, *E*^*Z*^ denotes the activation energy for the response, *k*_B_ is Boltzman constant, and *T* is absolute temperature. From the slopes of the fitted straight lines we obtain an activation energy of 0.64 eV for *σ*_*g*_ and that of 0.66 eV for *σ*_*gb*_. The comparable values of the activation energies of *σ*_*g*_ and *σ*_*gb*_ indicates that the relaxation and conduction process may be attributed to the same types of entities^22^.

### Electric modulus analysis

**Figure 8:**
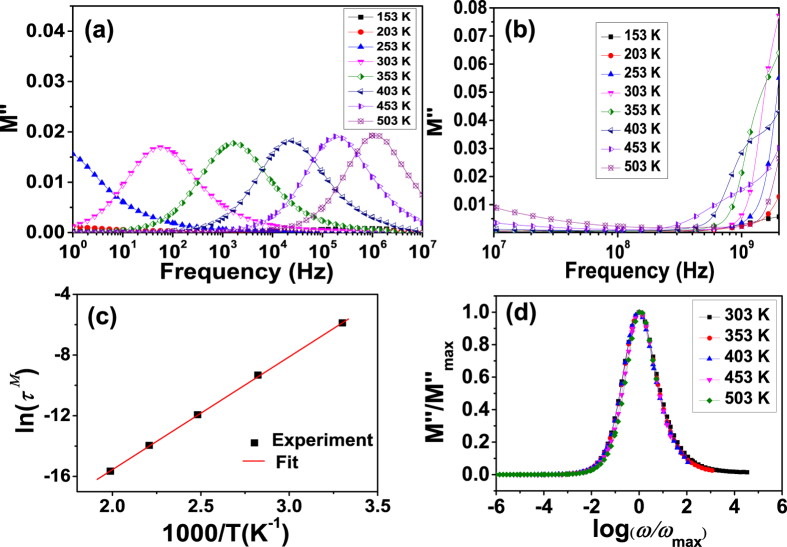
Temperature dependent imaginary part modulus *M*″ spectra (a) below 10^7^ Hz and (b) above 10^7^ Hz, (c) the temperature dependent relaxation times obtained from the peak frequencies of *M*″spectra above 303 K, (d) scaling behavior of the *M*″ spectra in plot (a) at temperatures above 303 K.

In the previous discussion, the dielectric relaxation has been analyzed in the formalisms of Z^*^. The modulus representation M^*^ = *M*′ − *iM*″is often used together with the impedance formalisms to distinguish the microscopic processes responsible for localized dielectric relaxations and long-range conductions^35^. Figure 8 shows the frequency dependence of imaginary part (*M*″) of the modulus M^*^ at different temperatures. Figure 8(a) shows that when the temperature is lower than 253 K, *M*″ is very small, indicating the freezing of dipoles in grain boundaries at low temperatures. When the temperature is increased above 303 K, a strong loss peak enters in the low frequency region with peak position shifting to higher frequency with increasing temperature. In the *M*″vs frequency spectra, the frequency region below the loss peak maximum determines the range in which charge carriers are mobile over long distances. At the frequency above peak maximum (high-frequency), the carriers are confined to potential wells, being mobile over short distances. The region where the peak occurs is indicative of the transition from long-range to short-range mobility with increase in frequency. This type of behavior of the modulus spectrum is suggestive of a temperature-dependent hopping type mechanism for electrical conduction (charge transport) in the system. The values of full-width-at-half-maxima calculated from the *M*″ loss spectra (*M*″ vs log *f*) are greater than 1.144 decades, indicating a non-Debye-type relaxation and a distribution of relaxation times^34^. The mean dielectric relaxation time *τ*^*M*^ is calculated using the relation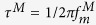, where 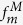 is peak frequency of *M*″. This relaxation time as a function of temperature is shown in the Fig. 8(c). It can be seen that the relaxation time *τ*^*M*^ can be well fitted with the Arrhenius relation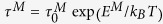, where 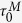 is the pre-exponential factor of the relaxation time. The activation energy *E*^*M*^ determined from the slope of the linear fit in the Arrhenius plot is 0.64 eV. It is interesting to note that the activation energy *E*^*Z*^ corresponding to *Z*″ spectra represent the localized conduction (i.e., dielectric relaxation) and that of *M*″ spectra represent nonlocalized conduction (i.e., long range conductivity)^37^. The comparable values of the activation energies of *E*^*Z*^ and *E*^*M*^ indicate that the relaxation and conduction processes may be attributed to the same types of charge carriers. This agree with the previous results in Fig. 7(d). Figure 8(b) shows that when the frequency is higher than 0.5 GHz, *M*″ increases greatly. This should be due to the resonance-dominated dielectric behavior at high frequencies as shown in Fig. 3. The *M*″ peaks are expected to appear above 2 GHz.

The above modulus spectra show that there is a distribution of relaxation times for dielectric relaxation at low frequencies. In order to confirm whether the distribution of relaxation time is temperature dependent or not, we plotted the *M*″ in scaled coordinates, i.e., 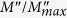 versus log(*ω*/*ω*_*max*_), where *ω*_*max*_ the loss peak frequency. As shown in Fig. 8(d), all peaks collapse into one master curve at different temperatures ranging from 303 K up to 503 K. It indicates that the dynamical processes are nearly temperature independent in low frequency region.

**Figure 9:**
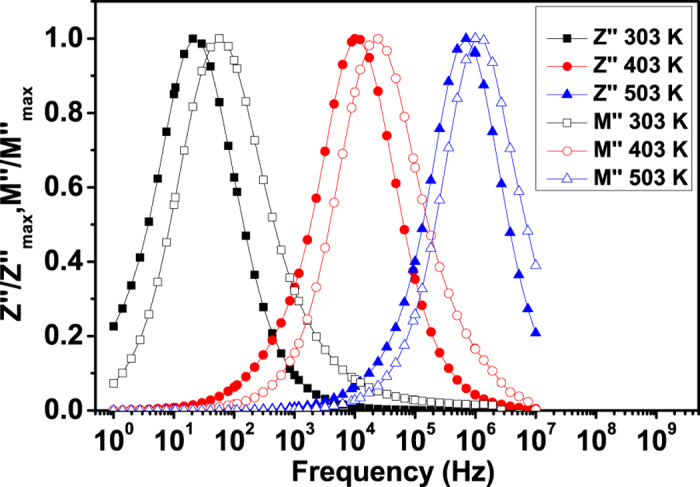
Normalized imaginary parts of electric modulus 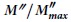 and impedance 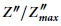 as functions of frequency at different temperatures for SCFO.

In the previous discussion, the dielectric relaxation has been analyzed in the formalisms of Z^*^ and M^*^ respectively. The combined plot of M^*^ and Z^*^ versus frequency is able to distinguish whether the short-range or long-range movement of charge carriers is dominant in a relaxation process^37^,^38^. The separation of peak frequencies between M^*^and Z^*^ indicates that the relaxation process is dominated by the short-range movement of charge carriers and departs from an ideal Debye-type behavior, while the frequencies coincidence suggests that long range movement of charge carriers is dominant^37^. The normalized functions of 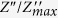 and 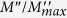 are shown in Fig. 9. The slight mismatch in the peak frequencies suggests a non-Debye-type behavior and coexisting of both long-range and localized relaxation. The mismatch between the *Z*″ and *M*″ becomes smaller with an increase in temperature, which indicates that the proportion of long-range charges movements increases with increasing temperature. This agrees with the previous results in Fig. 8 and the increase of both *σ*_*g*_ and *σ*_*gb*_ with increasing temperature as shown in Fig. 7. In order to mobilize the localized charge carriers, the aid of lattice oscillation is required. Under these circumstances, electrons are considered not to move by their own but by hopping motion activated by lattice oscillation^39^. Therefore, the increase of temperature can facilitate the long-range hopping movement of the charge carriers.

## Discussion

In summary, the dielectric properties of SCFO have been investigated as a function of temperature from 153 to 503 K between 1 and 2 GHz. The dielectric responses of SCFO are found to be frequency dependent and thermal activated. The relaxation-type dielectric behavior is observed to be dominating in the low frequency region and resonance-type dielectric behavior is found to be dominating above 10^8^ Hz. This frequency dependence of dielectric response is explained by the damped harmonic oscillator model with temperature dependent coefficients. The complex impedance plots show that the dielectric response of SCFO originates from both the grain and grain-boundary. The dielectric relaxation time decreases with increasing temperature in low frequency region and increases with increasing temperature above 10^8^ Hz. The imaginary part of impedance (*Z*″) and modulus (*M*″) spectra show that there is a distribution of relaxation times. The scaling behaviors of *Z*″ and *M*″ spectra further suggest that the distribution of relaxation times is temperature independent at low frequencies. The dielectric loss spectra at different temperatures have not shown a scaling behavior above 10^8^ Hz. A comparison between the *Z*″ and the *M*″ spectra indicates there are both short-range and long range motions of charge carriers with short-range motion dominating at low temperatures and long-range motion dominating at high temperatures. The above results indicate that the dielectric dispersion mechanism in SCFO is temperature independent at low frequencies and temperature dependent at high frequencies owing to the domination of resonance behavior.

## Methods

Polycrystalline SCFO hexaferrites were prepared with the solid state reaction method and an annealing procedure same as that in the previous work^9^. The starting high purity (>99.9%) powder Fe_2_O_3_, SrCO_3_, Co_3_O_4_ were weighed to prescribed proportion, mixed, ground and then annealed in air at 1000 °C for 16 h. The resulting powder was ground again, pressed into pellet, and then annealed in air at 1200 °C for 16 h (cooling down at a rate of 1 °C/min). Air annealing was used because it has been widely adopted by industry and the reported works to prepare the polycrystalline hexaferrites^1^,^3^,^9^,^12^,^13^,^14^,^20^,^21^,^22^. The phase of SCFO was checked by X-ray diffraction (XRD) on a Rigaku DMax 2000PC XRD diffractometer with Cu-K*а* radiation at room temperature. The grain structure of SCFO was measured from the broken interface of the sample with Scanning Electron Microscope (SEM, Hitachi, SU8010). The X-ray photoelectron spectroscopy characterization of SCFO was carried out on the PHI 5000 VersaProbe spectrometer with monochromatic Al K*a* radiation. The dielectric properties of SCFO were measured using a NOVOCONTROL high resolution dielectric analyzer (Concept 80) in the temperature range 153–503 K and the frequency range between 1 and 2 G Hz. Silver paint was used on the polished surfaces as electrodes. The complex impedance Z^*^ of the sample was obtained in the usual way, i.e., Z^*^ = V^*^/I^*^, where V^*^ and I^*^ are the applied voltage and the resulting current, respectively. The dielectric permittivity ε^*^ and modulus M^*^ spectra were obtained from the impedance spectra by using the relations below:


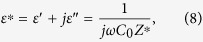



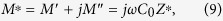


where *ω* is the angular frequency *ω* = 2*πf* and 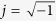. *C*_0_ = *ε*_0_*S*/*d* is the empty cell capacitance, where *S* is the sample area and *d* the sample thickness.

## Additional Information

**How to cite this article**: Tang, R. *et al.* Dielectric relaxation, resonance and scaling behaviors in Sr_3_Co_2_Fe_24_O_41_ hexaferrite. *Sci. Rep.* **5**, 13645; doi: 10.1038/srep13645 (2015).
